# Cutting Performance of Different Coated Micro End Mills in Machining of Ti-6Al-4V

**DOI:** 10.3390/mi9110568

**Published:** 2018-11-02

**Authors:** Zhiqiang Liang, Peng Gao, Xibin Wang, Shidi Li, Tianfeng Zhou, Junfeng Xiang

**Affiliations:** Key Laboratory of Fundamental Science for Advanced Machining, Beijing Institute of Technology, Beijing 100081, China; liangzhiqiang@bit.edu.cn (Z.L.); cutting0@bit.edu.cn (X.W.); 2120160347@bit.edu.cn (S.L.); zhoutf@bit.edu.cn (T.Z.); xjf2014@bit.edu.cn (J.X.)

**Keywords:** micro end milling, coating materials, tool wear, Ti-6Al-4V

## Abstract

Tool wear is a significant issue for the application of micro end mills. This can be significantly improved by coating materials on tool surfaces. This paper investigates the effects of different coating materials on tool wear in the micro milling of Ti-6Al-4V. A series of cutting experiments were conducted. The tool wear and workpiece surface morphology were investigated by analyzing the wear of the end flank surface and the total cutting edge. It was found that, without coating, serious tool wear and breakage occurred easily during milling. However, AlTiN-based and AlCrN-based coatings could highly reduce cutting edge chipping and flank wear. Specifically, The AlCrN-based coated mill presented less fracture resistance. For TiN coated micro end mill, only slight cutting edge chipping occurred. Compared with other types of tools, the AlTiN-based coated micro end mill could maximize tool life, bringing about an integrated cutting edges with the smallest surface roughness. In short, the AlTiN-based coating material is recommended for the micro end mill in the machining of Ti-6Al-4V.

## 1. Introduction

Micro end milling has many advantages in high-accuracy and high-efficiency machining and is capable of machining small complex structures with various materials at micro- and meso-scales compared with other micro machining methods [1,2]. However, tool life is a significant issue for the application of micro end mills. Problems such as cutting edge chipping, abrasive wear, and fatigue fracture easily occur [3], especially in the machining of difficult-to-cut aerospace alloy Ti-6Al-4V, resulting in very short tool life. Due to the low machinability, the application of coating materials is one of the crucially important methods for achieving a satisfactory tool life [4]. The hard coatings on cutting tools greatly helps to reduce tool wear and increase tool life. Therefore, applying hard coatings to micro end mills is a suitable method to extend tool life and improve the machining quality.

Many studies have shown that optimizing coating materials improves the cutting performance of micro tools. Ucun et al. [3] investigated the effects of coating materials on tool wear in micro milling of Inconel 718 super alloy and found that the micro end mills coated with AlTiN, TiAlN + AlCrN and AlCrN displayed better cutting performances compared to those coated with TiAlN + WC/C and diamond like carbon (DLC). Biermann et al. [5] reported TiAlN and AlCrN coated micro end mills presented good wear resistance, and better surface quality can be obtained by using AlTiN coated micro end mill in machining of austenitic stainless steel compared to CrN, TiN, AlCrN, AlTiN and TiAlN coated tools. Kumar et al. [6] reported the wear performance of TiAlN and Al_2_O_3_ + ZrN coated micro end mills is superior, and delamination is the principal wear mechanism of TiCN, TiSiN and Alumina (Al_2_O_3_) coated micro end mills in laser assisted micro-milling of hardened steel. Kang et al. [7] reported the CrAlSi(8.7 at%)N coating presented excellent microstructure, microhardness and tribological properties, and provided a better drilling performance in circuit board hole-drilling compared to CrN, CrAlN and CrSiN. Aramcharoen et al. [8] reported that TiN, CrN, TiAlN, TiCN and CrTiAlN coatings can reduce cutting edge chipping and edge radius wear of tools compared to uncoated cemented carbide micro end mills, and the TiN coating performs the best in micro milling of tool steel based on flank wear, edge radius wear, cutting edge chipping, surface finish and burr size. Aslantas et al. [9] reported the TiN and AlCrN coated micro end mills were less worn than uncoated and nano-crystalline diamond coated tools, and the changes in the diameters of TiN and AlCrN coated micro end mills were at a minimum. Xu et al. [10] reported the performance of Ti(C_7_N_3_)-based cermet micro end mill is better than WC micro end mill, and the wear mechanisms of the Ti(C_7_N_3_)-based cermet micro end mill are adhesion wear, micro chipping and diffusion wear in machining of aluminum alloy 2024. Torres et al. [11] found thin fine-grained diamond and nanocrystalline diamond coatings can improve the cutting performance and tool life of 0.3 mm diameter tungsten carbide micro end mills in slot milling of 6061-T6 aluminum. Therefore, the cutting performance and tool life of micro end mill can be effectively improved by using coating materials.

As for traditionally coated cutting tools, An et al. [12] reported micro-chipping and coating peeling were the primary tool failure modes for physical vapor deposition-AlTiN (PVD-AlTiN) coated cemented carbides, and serious abrasion wear and adhesive wear were the main wear modes in hard milling of 30Cr3. Jindal et al. [13] reported that TiAlN coated tools present the best cutting performance, followed by TiCN and TiN coated tools, and that TiAlN has higher hot hardness and oxidation resistance resulting in the coating’s higher abrasive and crater wear resistance. Kadirgama et al. [14] found that flank wear, notching, chipping, plastic lowering at cutting edge, catastrophic and wear at nose were the predominant tool failure for the PVD coated tools with TiAlN. Xue and Chen [15] reported that workpiece materials adhesion occurred on rake and flank faces in turning of nickel-based alloy GH4169 under wet conditions. Flank wear, crater on the rake face and notching are the dominant tool wear modes. Arndt and Kacsich [16] reported the AlTiN-Saturn coating presented an extraordinary performance in high speed cutting of hardened tool steel due to the high adhesion and ultrafine crystallinity and high oxidation resistance. Alvarez et al. [17] found a stratified multi built-up layer composed with TiOx is formed on the rake face of tools in the machining of Ti-6Al-4V alloy, and the initially built-up edge is mainly generated by mechanical adhesion mechanism. However, in micro end milling, the effects of coating materials on tool wear in the micro milling of Ti-6Al-4V are still unclear.

This paper aims to investigate the effects of different coating materials on cutting performance in the micro milling of Ti-6Al-4V. A series of cutting performance experiments were conducted on Ti-6Al-4V using micro end mills coated in different materials. To clarify the tool wear mechanisms, end flank wear length, the total cutting edge length reduction and workpiece surface morphology were also investigated.

## 2. Experimental Procedures

### 2.1. Fabrication of Coated Micro End Mills

**Figure 1:**
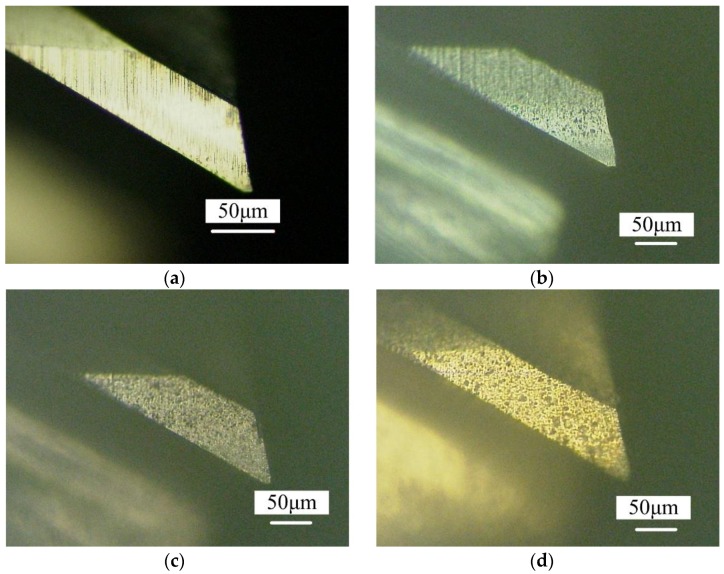
Flank face surface morphologies of differently coated micro end mills. (**a**) Uncoated micro end mill; (**b**) AlCrN-based coated micro end mill; (**c**) AlTiN-based coated micro end mill; (**d**) TiN coated micro end mill.

In the machining of difficult-to-cut material like titanium alloy Ti-6Al-4V, the micro end mill is prone to serious wear, with the cutting edge damage occurring early, thus significantly reducing the cutting performance. Coating the micro end mill is an effective way to solve this problem. This is because the coating material can increase the hardness and chemical stability of a surface and reduce the friction coefficient between the tool and workpiece. Micro end mills with two flutes and a diameter of 0.5 mm were used in the experiments. Table 1 shows the tool geometries. The mills were fabricated on the Makino Seiki six-axis CNC grinding machine (CNS7d, Makino Seiki Co., Ltd., Kanagawa, Japan). The substrate material of the mills was UF09 (IMC International Metal Working Engineering & Production Co., Ltd., Dalian, China), a fine cemented carbide with a grain size of 0.5 μm and 9% Co content. The coating materials were AlCrN-based (BALINIT^®^ ALNOVA), AlTiN-based (BALINIT^®^ LATUMA) and TiN (BALINIT^®^ BALINIT A) (Oerlikon Balzers, Co., Ltd., Balzers, Liechtenstein), respectively, and their characteristics are listed in Table 2. The surface coating thicknesses were about 1μm, and their effect on the tool geometry was negligible. The coated mills were measured using a Keyence 3D laser scanning microscope (VK-X100, Keyence Co., Ltd., Osaka, Japan) and scanning electron microscopy (SEM, FEI Quanta 650FEG). The cutting edge radii of the uncoated, AlCrN-based, AlTiN-based and TiN coated micro end mills were 0.41 μm, 0.90 μm, 1.2 μm and 1.5 μm, respectively. Figure 1 shows the surface morphologies of the flank faces coated with different materials. Obviously, the coated surfaces have different colors. Specifically, the AlCrN-based coating is gray, the AlTiN-based coating is gray black, and the TiN coating is golden-yellow.

**Figure 2:**
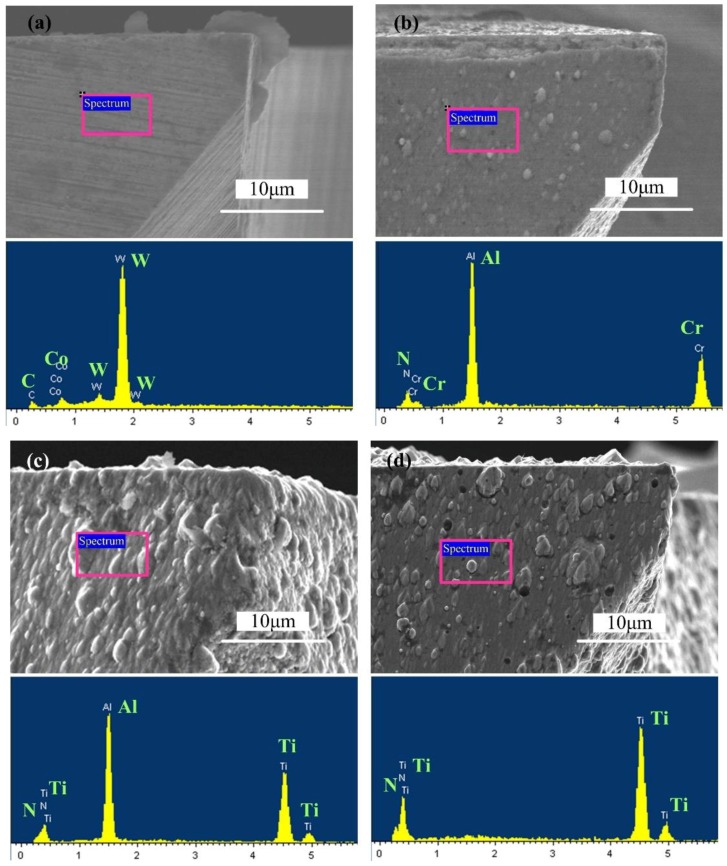
Surface morphologies and energy-dispersive spectroscopy (EDS) of differently coated micro end mill flank faces. (**a**) Uncoated micro end mill; (**b**) AlCrN-based coated micro end mill; (**c**) AlTiN-based coated micro end mill; (**d**) TiN coated micro end mill.

Surface morphologies and energy spectrum analyses of the coated mills’ flank faces are shown in Figure 2. It can be seen that the flank faces of coated micro end mills are covered with coating materials. The composition of the uncoated micro end mill is mainly composed of W and Co elements. The C element signal identified in the energy spectrum may be caused by residual grinding oil on the tool. The energy spectrum of the AlCrN-based coated micro end mill is mainly composed of Al, N and Cr elements, while the energy spectrum of the AlTiN-based coating is mainly composed of Al, N and Ti elements. The tool with the TiN coating is mainly composed of Ti and N elements. Thus, the surfaces of coated micro end mills are mainly composed of coating elements. The W and Co elements of the cemented carbides are not found in the energy spectrum of the coated mills, indicating that the surfaces of coated mills are complete and no partial coating occurs.

### 2.2. Cutting Performance Experiments

The cutting experiments were conducted on a five-axis machining center (DMU80 monoBLOCK by DMG MORI Co., Ltd., Nagoya, Japan). Ti-6Al-4V was selected as the workpiece material. The cutting parameters of the experiments are listed in Table 3. After machining, the micro end mills and machined workpieces were cleaned by an ultrasonic cleaner before being measured. The surface morphologies of the tools and machined microgrooves were measured using the 3D laser scanning microscope, and the surface roughness Sa of the microgroove bottoms measured by white light interferometer (Talysurf CCI Lite by Taylor Hobson Co., Ltd., Leicester, UK). The wear morphologies of the micro end mills were also measured using SEM equipped with energy-dispersive spectroscopy (EDS). The reductions of tool end teeth flank wear length and total cutting edge length were also measured per machining length of 80 mm by the 3D laser scanning microscope.

## 3. Results and Discussion

### 3.1. Tool Wear

**Figure 3:**
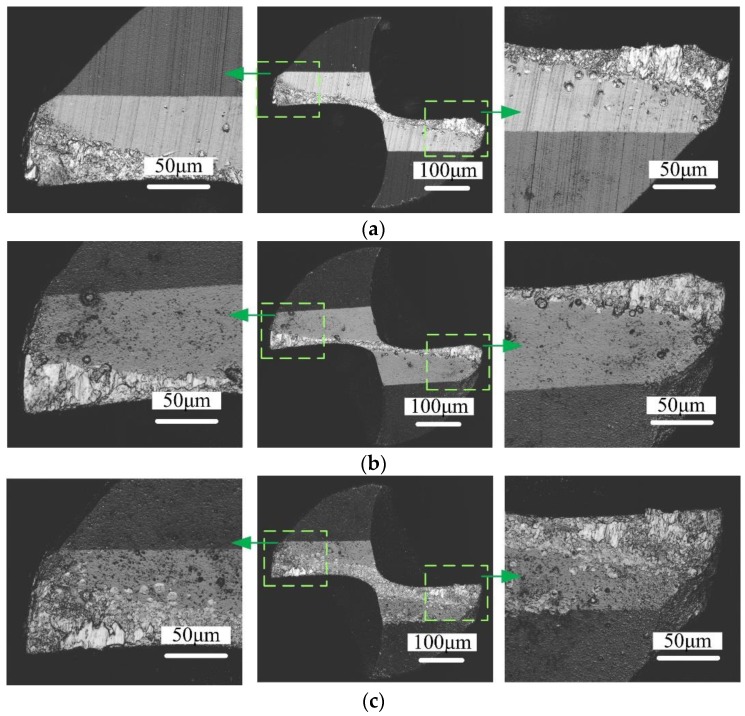

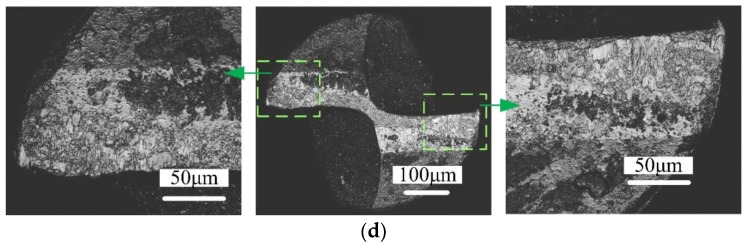
Surface morphologies end teeth flank faces of micro end mills with different coating materials (*v*_c_ = 20 m/min, *f*_z_ = 2 μm/z, *a*_p_ = 50 μm, 640 mm). (**a**) Uncoated micro end mill; (**b**) AlCrN-based coated micro end mill; (**c**) AlTiN-based coated micro end mill; (**d**) TiN coated micro end mill.

The surface morphologies of micro end mill end teeth flank faces with different coatings after a cutting length of 640 mm are shown in Figure 3. It can be seen that the loss of sharpness and the geometry deterioration of mill appear due to tool wear. The wear of the uncoated micro end mill tool is severe, and cutting edge chipping occurs. The AlCrN-based coated micro end mill has the highest wear resistance, resulting in the shortest end teeth wear length. However, the AlCrN-based coated micro end mill is also accompanied by cutting edge chipping. Meanwhile, the cutting edge of AlTiN-based coated micro end mill is complete, and the cutting edge rounding is less. Its cutting edge is intact, and no cutting edge chipping occurs. However, a titanium alloy adhesion layer is generated on the end teeth flank face of the tool. For TiN coated micro end mill, although minor chipping of the cutting edges occurs, the end teeth flank faces of the tool are adhered with a large amount of titanium alloy material. Thus, the TiN coating material presents stronger adhesion characteristics to the titanium alloy.

**Figure 4:**
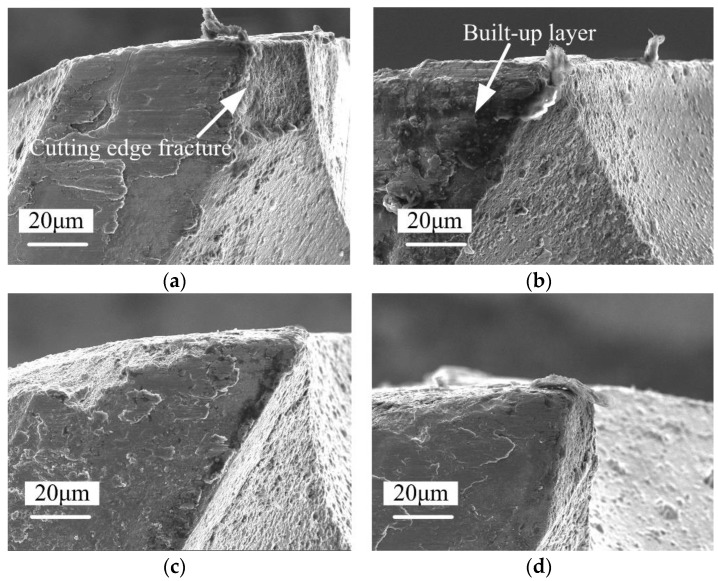
Flank face surface wear morphologies of micro end mills with different coating materials (*v*_c_ = 20 m/min, *f*_z_ = 2 μm/z, *a*_p_ = 50 μm, 640 mm). (**a**) Uncoated micro end mill; (**b**) AlCrN-based coated micro end mill; (**c**) AlTiN-based coated micro end mill; (**d**) TiN coated micro end mill.

The flank face wear morphologies of differently coated micro end mills after a cutting length of 640 mm are shown in Figure 4. It can be seen that the Ti-6Al-4V workpiece material adheres to the flank face of the micro end mills, forming a built-up layer. The uncoated micro end mill cutting edges exhibit breakage. Cutting edge rounding of all micro end mills is also observed, which indicates abrasive wear gradually occurs on every tool. Also, workpiece material is adhered on the tool surface, and a multilayer is formed onto the flank face of the micro end mill. The rounding of the cutting edges indicates the gradual wear of the tool. The AlTiN-based coated micro end mill shows a more complete cutting edge, and the cutting edge rounding radius is smaller.

**Figure 5:**
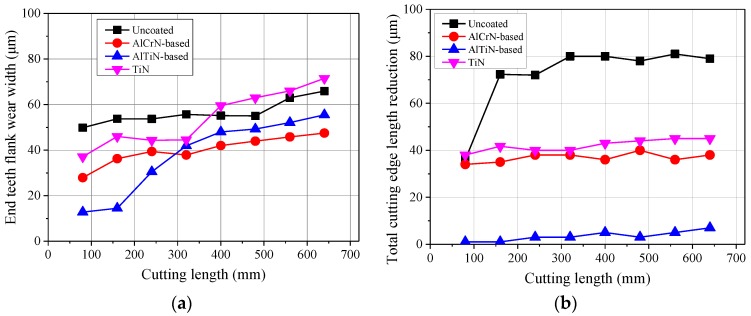
End teeth flank wear length and total cutting edge length reduction of micro end mills with different coatings (*v*_c_ = 20 m/min, *f*_z_ = 2 μm/z, *a*_p_ = 50 μm). (**a**) End teeth flank wear length; (**b**) Total cutting edge length reduction.

To evaluate the effects of differently coated micro end mills on cutting performance, the end teeth flank wear length and total cutting edge length reduction were investigated. The corresponding results are shown in Figure 5. It can be seen that the end teeth flank wear length of the uncoated micro end mill increases rapidly with the increase of cutting length. The end teeth flank wear length of AlCrN-based and TiN coated micro end mills are smaller than that of uncoated tools. The TiN and AlCrN coated micro end mills are less worn than the uncoated one, and these conclusions are also in accordance with the literature [9]. The uncoated micro end mill has more serious tool breakage, and the total cutting edge length reduction of this micro end mill has also increased significantly with the increase of cutting length. Compared with the AlTiN-based coated tool, the total cutting edge length reduction of the AlCrN-based coated mill is larger, which suggests less fracture resistance. Compared with other tools, the total cutting edge length reduction of the AlTiN-based coated tool is the smallest and slightly increases with the increase of cutting length.

### 3.2. Tool Wear Mechanisms

**Figure 6:**
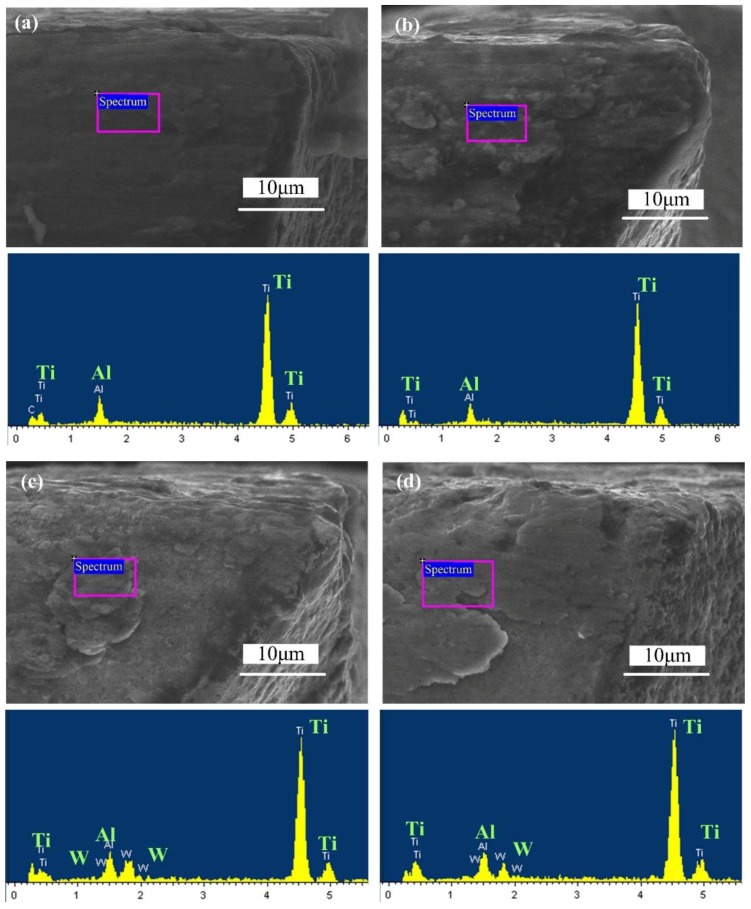
Surface wear morphologies and energy-dispersive spectroscopy (EDS) of differently coated micro end mill flank faces (*v*_c_ = 20 m/min, *f*_z_ = 2 μm/z, *a*_p_ = 50 μm, 640 mm). (**a**) Uncoated micro end mill; (**b**) AlCrN-based coated micro end mill; (**c**) AlTiN-based coated micro end mill; (**d**) TiN coated micro end mill.

Figure 6 shows the surface wear morphologies and EDS of differently coated micro end mill flank faces after a cutting length of 640 mm. It is clear that the rake and flank faces of all tools are being adhered to by Ti-6Al-4V workpiece material. Cutting edge rounding is also observed, indicating the gradual abrasive wear of the tools. The W and Co elements of cemented carbides are not found in the energy spectrum of the uncoated micro end mill flank face since the flank face is covered by Ti-6Al-4V workpiece material. The energy spectrum of the AlCrN-based coated micro end mill is mainly composed of Ti and Al elements. The energy spectrum of the AlTiN-based coated tool is similar to the Ti-6Al-4V workpiece material. This indicates that the tool material has also been peeled off, and the flank face of the AlCrN-based tool is being adhered to by workpiece material. Similarly, the energy spectrum of the AlTiN-based coated micro end mill is mainly composed of Ti, Al and W elements, and the N element in the AlTiN-based coating is not found. Additionally, the W element in the cemented carbide bulk material is found. This indicates the AlTiN-based coating material of the tool surface is peeled off. However, the cutting edge of AlTiN-based coated tool is more complete, with little cutting edge chipping appearing on the cutting edge of the tool. The coating on the tool surface has been completely removed, but the cutting edge is still intact. At the same time, the energy spectrum of the micro end mill flank face with a TiN coating is mainly composed of elements from the workpiece material, which indicates that the TiN coating materials are peeling off and the flank face is being adhered to by workpiece material. It can be concluded that the tool failure modes of all the coated micro end mills are cutting edge chipping and coating delamination. The dominant wear modes are severe adhesive wear and abrasion wear on the tool flank and rake faces.

In micro milling, the cutting edges of tools suffer cyclic flexural and compressive stress, thus the stress concentrates in the cutting edges. Dislocation behavior occurs in coating material, resulting in micro crack nucleation. Under the action of alternating stress, the initiation of micro cracks begins, and then the micro cracks propagate, resulting in cutting edge chipping. The cutting edge chipping causes the reduction of the total cutting edge length, leading to a decrease in microgroove width. Moreover, with an increase in cutting length, the cutting edge radius becomes larger due to micro chipping of the cutting edge, and then plowing behavior prevails. Under high pressure, workpiece material tends to adhere to the surface of the micro end mill in the contact zone, and the workpiece material welds to the tool surface forming an adhesion layer. A built-up edge and built-up layer are also generated as the result of the high cutting pressure and the high chemical affinity with Ti-6Al-4V. The coatings are pulled off and dragged by the adjacent Ti-6Al-4V, and the dislocation, plastic deformation and breakage of the coatings occur at the cutting contact. Moreover, the contact of micro milling is at the microscale, and adsorption energy becomes the key factor dominating tool adhesion at the microscale. The formation mechanism of a built-up edge and built-up layer depends on both tool and workpiece materials [17]. The hardness of the AlCrN coating is higher than the AlTiN coating (Table 2); therefore, the AlCrN-based coated tool has more wear resistance after a cutting length of 320 mm. The Cr_x_Al_y_N coated cutting tools may have sufficient potential to become a machining alternative compared to the AlTiN coating. However, the affixed Cr_x_Al_y_N coating has a poor machining performance due to its brittle structure or high coefficient of friction [18]. Thus, compared to the AlTiN-based coated tool, the AlCrN-based coated tool presents less fracture resistance. The TiN coating has the lowest hardness and maximum service temperature compared to AlCrN-based and AlTiN-based coatings. So the TiN coated micro end mill presents stronger adhesion characteristics to the titanium alloy workpiece material. For the TiN coated micro end mill, although minor chipping of the cutting edge occurs, the end teeth flank faces of the tool is more often adhered with a large amount of titanium alloy material. For AlTiN coating, Al element is added to the TiN based composition, providing not only a greater hardness, but also a remarkable improvement in inertness and high temperature strength [4]. The superior cutting performance of AlTiN coated tools over those coated with TiN can be partly attributed to the solid solution strengthening effect of Al in the TiN lattice [13].

### 3.3. Surface Morphologies and Surface Roughness of Machined Microgrooves

**Figure 7:**
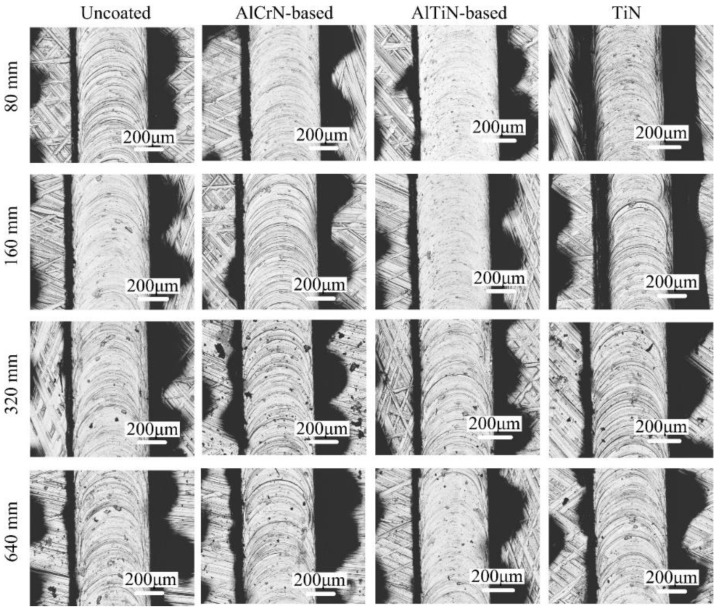
Surface morphologies of microgrooves machined with coated micro end mills.

The surface morphologies of microgrooves machined with differently coated micro end mills are shown in Figure 7. It can be seen that, with the uncoated mill, a serious plowing phenomenon occurs on the surface of microgrooves only after a machining length of 80 mm. The microgrooves machined with the AlCrN-based coated mill also generate a serious plowing phenomenon at the machining length of 160 mm. However, the bottom surfaces of microgrooves machined with the AlTiN-based coated micro end mill are smoother, and no obvious plowing phenomenon occurs. Due to the high hardness, the tool presents less wear, good surface integrity and a sharp cutting edge. The serious chip adhesion is found on the surface of the microgrooves machined with the TiN coated micro end mill, and severe plowing is generated on the machined surface. Due to the strong affinity between TiN coating material and titanium alloy, the titanium alloy material is easily attached to the cutting edge, which leads to surface plowing and worsens surface quality.

**Figure 8:**
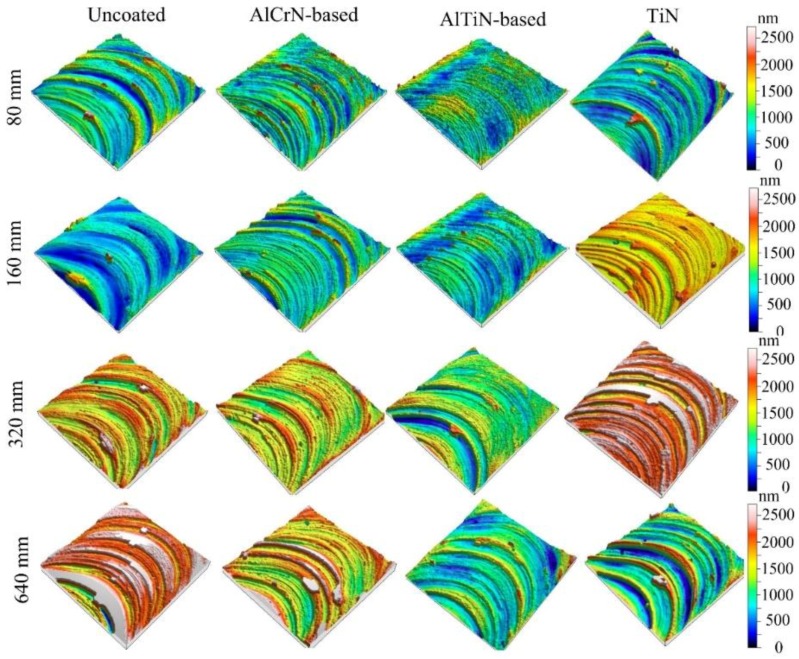
Surface morphologies of microgroove bottoms machined with coated micro end mills.

The surface morphologies of microgroove bottoms are shown in Figure 8. Clearly, the surface morphologies of the microgrooves processed with differently coated micro end mills are quite different. For the uncoated and TiN coated micro end mills, deeper plowing concave and convex surfaces are generated in the bottom surface. Moreover, serious surface plowing occurs in the bottom of microgrooves that are machined by the AlCrN-based coated micro end mill. However, machining with the AlTiN-based coated mill is smoother. The surface quality is first uniform, and then becomes non-uniform. With the increase in cutting length, the mill cutting edge radius increases resulting in a more prevalent size effect. Due to the size effect of micro milling, high temperature and stress is generated in the cutting zone. The Ti-6Al-4V material then is compressed following plastic deformation. Finally, it adheres to the tools and the machined surface.

**Figure 9:**
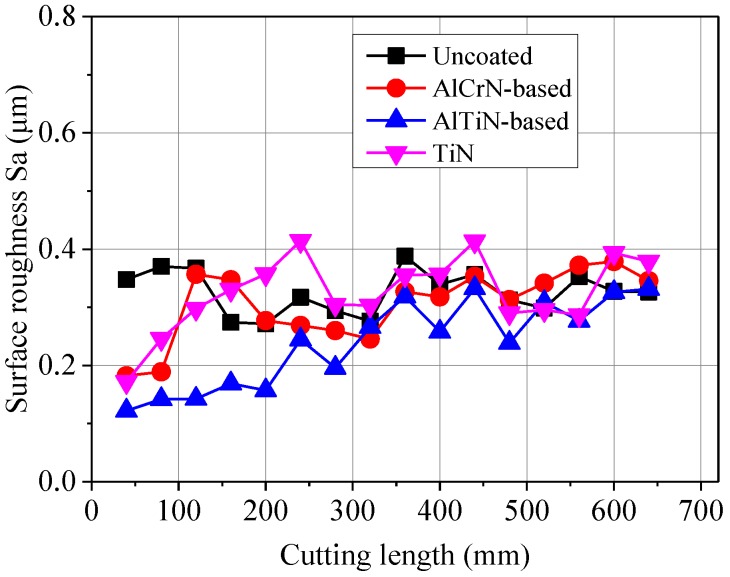
Surface roughness of microgroove bottoms with increased cutting length.

Figure 9 shows the variation of surface roughness Sa of the microgroove bottoms with increases in cutting length. It can be seen that the surface roughness of Ti-6Al-4V machined by the AlTiN-based coated mill is the lowest when compared with the other three types of coatings. With an increase in cutting length, the micro end mill tool wear becomes serious, and the surface roughness Sa of the machined microgroove slightly increases. The built-up edge and built-up layer can generate additional tool surface, which will increase the friction extrusion between the workpiece and micro tool, leading to the deterioration of the machined surface. Moreover, the random removal of a built-up edge also causes fluctuation of the surface roughness.

## 4. Conclusions

This paper investigates the effects of coating materials on the cutting performance of micro end mills. A series of cutting experiments were conducted on Ti-6Al-4V with different materials. The end flank wear length and the total cutting edge length reduction were also investigated. Based on this work, the following conclusions were obtained:

(1) The AlTiN-based and AlCrN-based coatings can lead to the reduction of cutting edge chipping and tool wear length compared to an uncoated micro end mill.
(2) Compared to uncoated, AlCrN-based and TiN coated tools, the AlTiN-based coated micro end mill has the longest service life. Its cutting edge remains intact after a longer cutting distance. Moreover, the surface roughness of the machined Ti-6Al-4V is the lowest for this coating when compared with the other three types of coating. Thus, the AlTiN-based coated material is suitable for the micro end mill in the machining of Ti-6Al-4V.
(3) The tool failure modes of all coated micro end mills are cutting chipping at cutting edge and coating delamination. The dominant wear modes are severe adhesive wear and abrasion wear on the tool flank face and rake face.

## Figures and Tables

**Table 1:** Tool geometries of the micro end mills.

Parameters	Helical Angle (°)	Rake Angle (°)	Relief Angle (°)	Helical Length (mm)
Values	30	0	3	1.5

**Table 2:** Characteristics of the different coating materials.

Coating Types	Hardness H_IT_ (GPa)	Max. Service Temp. (°C)	Coating Color	Advantages
AlCrN-based	38 ± 3	>1100	Light grey	High abrasion-resistance, high thermal shock stability, increased oxidation resistance
AlTiN-based	35 ± 3	1000	Grey	Superior oxidation resistance and hot hardness, optimal crater wear resistance
TiN	30 ± 3	600	Golden-yellow	Effective reduction of abrasive and adhesive wear

**Table 3:** Experiment cutting parameters.

Parameters	Cutting Speed *v*_c_ (m/min)	Feed Rate *f*_z_ (μm/z)	Depth of Cut *a*_p_ (μm)	Coolant
Values	20	2	50	dry
